# USP7 inhibition induces apoptosis in glioblastoma by enhancing ubiquitination of ARF4

**DOI:** 10.1186/s12935-021-02208-z

**Published:** 2021-09-23

**Authors:** Tingzheng Pan, Xuetao Li, Yanyan Li, Zhennan Tao, Hui Yao, Yue Wu, Guangliang Chen, Kai Zhang, Youxin Zhou, Yulun Huang

**Affiliations:** 1 Neurosurgery & Brain and Nerve Research Laboratory, The First Affiliated Hospital of Soochow University, Jiangsu Suzhou, People’s Republic of China; 2 Department of Neurosurgery, Dushu Lake Hospital Affiliated of Soochow University, Jiangsu Suzhou, People’s Republic of China

**Keywords:** GBM, USP7, Ubiquitination, ARF4, Apoptosis

## Abstract

**Background:**

Glioblastomas (GBMs) are grade IV central nervous system tumors characterized by a poor prognosis and a short median overall survival. Effective induction of GBM cell death is difficult because the GBM cell population is genetically unstable, resistant to chemotherapy and highly angiogenic. In recent studies, ubiquitin-specific protease 7 (USP7) is shown to scavenge ubiquitin from oncogenic protein substrates, so effective inhibition of USP7 may be a potential key treatment for GBM.

**Methods:**

Immunohistochemistry and western blotting were used to detect the expression of USP7 in GBM tissues. In vitro apoptosis assay of USP7 inhibition was performed by western blotting, immunofluorescence, and flow cytometry. Anti-apoptotic substrates of USP7 were defined by Co-IP and TMT proteomics. Western blotting and IP were used to verify the relationship between USP7 and its substrate. In an in vivo experiment using an intracranial xenograft model in nude mice was constructed to assess the therapeutic effect of target USP7.

**Results:**

Immunohistochemistry and western blotting confirmed that USP7 was significantly upregulated in glioblastoma samples. In in vitro experiments, inhibition of USP7 in GBM induced significant apoptosis. Co-IP and TMT proteomics identified a key anti-apoptotic substrate of USP7, ADP-ribosylation factor 4 (ARF4). Western blotting and IP confirmed that USP7 interacted directly with ARF4 and catalyzed the removal of the K48-linked polyubiquitinated chain that binded to ARF4. In addition, in vivo experiments revealed that USP7 inhibition significantly suppressed tumor growth and promoted the expression of apoptotic genes.

**Conclusions:**

Targeted inhibition of USP7 enhances the ubiquitination of ARF4 and ultimately mediates the apoptosis of GBM cells. In a clinical sense, P5091 as a novel specific inhibitor of USP7 may be an effective approach for the treatment of GBM.

**Supplementary Information:**

The online version contains supplementary material available at 10.1186/s12935-021-02208-z.

## Background

Glioblastomas (GBMs) are grade IV central nervous system tumors characterized by a poor prognosis [1] and a median overall survival that remained at around 15 months for decades [2]. Comprehensive treatments for glioblastoma, such as maximal surgical resection, chemotherapy, and radiation therapy, do not benefit all patients equally and their adverse effects can seriously affect quality of life [3, 4]. The GBM cell population is genetically unstable, resistant to chemotherapy and highly angiogenic [5]. Therefore, individualized treatment targeting several abnormal epigenetic functions in GBM should be considered as a potentially valuable approach for these patients.

Ubiquitination is a post-translational modification in which ubiquitin (Ub) molecules are sequentially bound to lysine residues of substrate proteins [6]. Substrate proteins are stabilized and function under the combined regulation of the ubiquitin-proteasome system (UPS) and deubiquitinating enzymes (DUBs) [7]. The regulation of most key proteins determines cell proliferation, migration and apoptosis [8]. The pathways being regulated have different results depending on the ubiquitination site. Ub molecules contain seven lysine residues (Lys6, Lys11, Lys27, Lys29, Lys33, Lys48, and Lys63) and different types and lengths of Ub chains can be formed. [9]. Tripartite motif 22 (TRIM22) can regulate the NF-κB signaling pathway through both the k48-mediated proteasome pathway and the k63-mediated phosphorylation pathway [10]. In addition, temozolomide resistance and radioresistance in GBM are also regulated by ubiquitination and deubiquitination [11, 12]. Ubiquitin-specific proteases (USPs), with more than 60 members, are the largest subfamily of DUBs. Ubiquitin-specific protease 7 (USP7; also known as Herpesvirus-associated ubiquitin-specific protease, HAUSP) is a cysteine protease originally identified as a binding partner for the herpes simplex viral (HSV) protein infected cell protein 0 (ICP0/Vmw110) [13]. USP7 is now considered to be an oncoprotein in many cancers due to the regulating the stability of other oncogenic proteins and inhibiting the nuclear translocation effects of oncogenic proteins [14, 15]. Targeting USP7 in GBM is therefore expected to cause cell death for therapeutic purposes.

GBM cell death can be achieved by promoting apoptosis [16]. According to previous studies, GBM apoptosis is influenced by spindle morphology, but also by endomitochondria, endoplasmic reticulum and redox status [17–19]. The Bcl-2 family is considered to be one of the most prominent apoptotic pathways because of its association with mitochondrial dysfunction and the release of intermembrane proteins, such as cytochrome c, that activate caspases [20]. ADP-ribosylation factor 4 (ARF4) has recently been identified as a novel anti-apoptotic factor involved in the Bcl-2 family-related apoptotic pathway [21]. ADP-ribosylation factors (ARFs) are 20 kDa small guanine nucleotide-binding proteins belonging to the Ras superfamily of small G proteins [22]. There are three classes of ARF family members: class I (ARF1, -2, and − 3), class II (ARF4 and − 5), and class III (ARF6). Class I and class III ARFs are involved in the transport of intracellular plasma membrane systems [23]. In contrast, ARF4 (class II) is defined as an anti-apoptotic protein that acts as a BAX inhibitor [24, 25]. However, the molecular mechanism underlying the stability of ARF4 in GBM is still unclear.

Here, we explored the apoptotic phenotype and triggering mechanisms following inhibition of USP7 in GBM in three ways. For experiments with clinical samples, we directly measured USP7 expression in clinical GBM tissue to enhance the clinical significance of the study. For in vitro experiments, we first assessed changes in phenotype following pharmacological inhibition and genetic silencing of USP7. We then searched for substrate proteins of USP7 in the protein pool by means of mass spectrometry and proteomics. Given the deubiquitinating enzymatic nature of USP7, we designed a series of in vitro ubiquitination experiments. For in vivo experiments, we observed the therapeutic effect of intracranial injections of USP7 inhibitors in a nude mouse intracranial xenograft model.

## Materials and methods

### Brain tissue specimens and cell culture lines

Human GBM tissue samples and normal brain contusion tissues were obtained from the First Affiliated Hospital of Soochow University, Suzhou, China. This study was approved by the Ethics Committee of Soochow University. Human SHG-140 cell lines were obtained from the Department of Neurosurgery & Brain and Nerve Research Laboratory, The First Affiliated Hospital of Soochow University, Suzhou, China, after primary culture and identification by STR [26]. T98G cell lines were obtained from the Cell Bank of the Chinese Academy of Sciences (Shanghai, China). Cells were cultured in DMEM (Gibco, USA) containing 10 % fetal bovine serum (FBS).

### Antibodies

Anti-USP7 (CST#4833), anti-BAX (CST#89,477), anti-CLEAVED-CASPASE 3 (CST#9664), anti-β-tubulin (CST#2146), and anti-Ub (CST#3933) were purchased from CST, USA. Anti-Bcl-2 (Ab692), anti-ARF4 (Ab171746), anti-K48 linkage-specific Ub (ab140601), and anti-K63 linkage-specific Ub (ab179434) were obtained from Abcam, UK. Horseradish peroxidase-labeled goat anti-mouse IgG and goat anti-rabbit IgG were purchased from ZSGB-Bio (China). P5091 (s7132), CHX (s7418), and MG132 (s2619) were purchased from Selleck (USA).

### Lentiviral and siRNA transfection

GeneChem (China) designed two shRNAs lentivirus against USP7, shUSP7-1: 5’ -UGUAUCUAUUGACUGCCCUTT- 3’ and shUSP7-2: 5’ -UGGAUUUGUGGUUACGUUACUC-3’, and an ARF4 overexpression lentivirus. The constructed lentiviral vector was transfected into cells followed by puromycin intervention, and the transfection efficiency was identified by western blotting analysis to screen for stably transfected cell lines. Sangon Biotech (China) designed two siRNAs against ARF4, siARF4-1: 5’ -GCAAGACAACCAUUCUGUAUATT- 3’ and siARF4-2: 5’ - CCAUCAGUGAAAUGACAGAUATT- 3’. Lipofectamine 3000 was used for co-transfection for 8 h, and transfection efficiency was investigated after transfection.

### Immunohistochemistry(IHC)

Tissues were paraffin-embedded and sectioned for immunostaining. Slides were dewaxed in xylene, rehydrated, followed by quenching of endogenous peroxidase activity with 0.3 % hydrogen peroxide and non-specific proteins were blocked with 5 % goat serum (Solarbio, China). Sections were incubated with primary antibody at 4◦ C overnight. Next, slides were incubated with ABC peroxidase and diaminobenzidine (ZSGBBio), followed by nuclear staining with Mayer hematoxylin solution (Solarbio) counterstain. For hematoxylin and eosin (H&E) staining, slides were passed through nuclear staining and subsequently re-stained using the H&E kit (Solarbio). Images were acquired using an inverted microscope (Olympus, Japan).

### Western blotting and immunofluorescence analyses

After induction, cells were collected in lysis buffer, incubated on ice for 30 min, and centrifuged at 12 000 × *g* at 4 °C for 10 min. The supernatant was collected, and the protein concentration determined. Equal amounts of protein samples were subjected to 8–12 % SDS-PAGE, transferred to PVDF membranes, and incubated at room temperature or 1 h using 5 % skim milk with primary antibody overnight. Membranes were washed with PBST buffer for 30 min and then incubated with a secondary antibody for exposure. For immunofluorescence, cells induced on slides were fixed with 4 % paraformaldehyde and permeabilized with 0.5 % Triton for 15 min. Slides were incubated at room temperature with 5 % BSA for 1 h, BSA was discarded, and mouse anti-BCL2 antibody and rabbit anti-CLEAVED-CASPASE3 antibody were added for overnight incubation. The next day, the primary antibody was discarded and the slides were incubated with secondary antibodies, Alexa Fluor 594-labeled goat anti-rabbit or Alexa Fluor 488-labeled goat anti-mouse for 1 h at 37 °C in a light-proof oven at a constant temperature. Fluorescence microscopy and image acquisition were performed under light-proof conditions.

### Cell viability assay (CCK-8 assay)

P5091 were added to 96-well plates containing cell suspensions in a total volume of 100 µl. After induction for 24, 48, and 72 h, 10 µl of CCK-8 reagent (Dojindo, Japan) was added and incubated at 37 °C for 2 h in a constant temperature incubator. Cell viability was evaluated by measuring the difference in optical density values at 450 nm using an enzymatic standard.

### Annexin V-FITC/PI flow cytometry for apoptosis detection

After apoptosis induction, cells were digested with 0.25 % trypsin and centrifuged at 2 000 × *g* for 3 min at 4 °C, resuspended in pre-chilled PBS, and centrifuged again at 2 000 × *g* for 3 min at 4 °C. The supernatant was discarded, and Annexin V-FITC, PI, and binding buffer (BD, USA) was added to the cell precipitate. After incubation for 10 min at 4 °C protected from light, cell fluorescence was detected by flow cytometry. For data analysis, Annexin V-FITC + PI- subpopulation was considered early-stage apoptosis and Annexin V-FITC + PI + was late-stage apoptosis or necrosis. Cell proportion comparisons between treatment or groups were done in both early- and late-stage subpopulation separately and in total.

### Tandem mass tags (TMT) proteomics analysis

Total protein samples were extracted for concentration determination by SDS-PAGE, trypsin digestion, and TMT peptide labeling. Equal amounts of labeled samples were mixed and separated by chromatography. Samples were loaded onto a pre-column Acclaim PepMap100 (RP-C18, Thermo Fisher) at a flow rate of 300 nl/min, and then separated on an analytical column. Finally, samples were analyzed by LC-MS/MS.

### Immunoprecipitation (IP)

For Co-IP and ubiquitination-related experiments, anti-ARF4 was added to the cell protein extracts, and for combined Co-IP and IP-MS experiments, anti-USP7 was added to the extracts. The mixture was then incubated with protein A/G magnetic beads overnight at 4 °C. Precipitates were washed three times with lysis buffer, boiled in 1x SDS sample buffer for 5 min, and proteins resolved by SDS-PAGE on 8–12 % gels. Immunoblot detection was performed using appropriate antibodies.

### Mass spectrometry (MS)

For MS, immunoprecipitates were resolved on SDS-PAGE denaturing gels, stained with Coomassie Blue, and then analyzed by MS. MS was performed with a Q-Exactive mass spectrometer (Thermo Scientific).

### Nude mouse intracranial xenograft model

Female BALB/c nude mice (4–5 weeks, 15–17 g) were purchased from the Animal Center of the Institute of Oncology, Chinese Academy of Medical Sciences (Beijing, China). A total of 5 × 10^4^ SHG-140 cells with luciferase-encoding lentivirus (GeneChem, Shanghai, China) were stereotactically injected into mice (six per group). P5091 was dissolved in 20 % DMSO, 40 % PEG-300, and 40 % PBS. Seven days after implantation, mice were injected intraperitoneally with equal doses of 10 mg/kg/day, 5 mg/kg/day of P5091, or PBS 2 days per week (a total of 3 weeks) during the survival period. Intracranial tumor size was assessed, and radiance values recorded on days 7, 14, and 28 using the IVIS Spectral Real-Time Imaging System (Blandford, USA). Live mouse brains were removed, fixed in 4 % paraformaldehyde, embedded in paraffin, and subjected to HE and IHC. Animal studies were performed according to internationally accepted norms and national regulations.

### Statistical analysis

Statistical analyses were performed with SPSS 16.0 or GraphPad Prism 8.2.1 software. Bar statistical plots were expressed by the mean standard deviation of at least three times the number of experimental replicates. Differences between two groups were assessed by Student’s t-test, and differences between multiple groups were tested by one-way analysis of variance (ANOVA) followed by Tukey’s post hoc test. Bars are expressed as mean ± s.e.m. The Kaplan-Meier Survival analysis was used to estimate the prognostic value of each genes and count the survival time of the nude mice. Statistical significance is shown at ^#^P = NS, *P < 0.05,**P < 0.01,***P < 0.001, or ****P < 0.0001.

## Results

### USP7 is highly expressed in GBM cells and its inhibition induces apoptosis

**Fig. 1:**
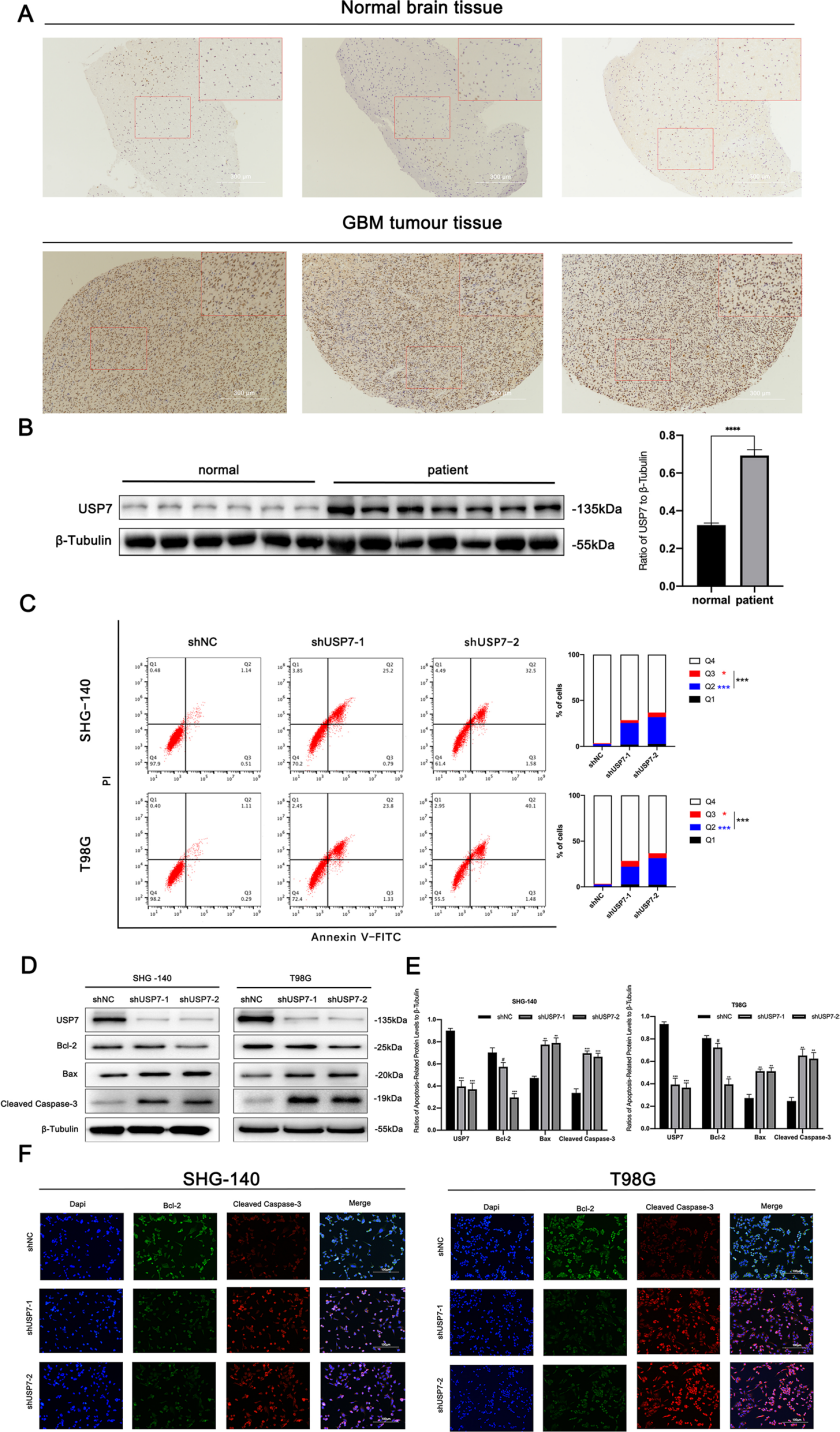
USP7 is highly expressed in GBM cells and its inhibition induces apoptosis. ** A** Immunohistochemical staining of USP7 in human glioblastoma and normal brain tissue samples. Scale bar, 300 μm. **B** Western blotting analysis of USP7 protein levels in primary glioblastoma tissue samples and normal brain tissue samples, n > 6. **C** Apoptosis of SHG-140 and T98G cells treated by shUSP7s was measured by flow cytometry, n = 3. Q3 (Annexin V-FITC + PI-) subpopulation was considered early-stage apoptosis and Q2 (Annexin V-FITC + PI+) was late-stage apoptosis or necrosis. Cell proportion comparisons between NC and shUSP7 groups were done in both early- and late-stage subpopulation separately and in total. **D**, **E**. SHG-140 and T98G cells were treated with two different shUSP7s for 24 h. The changes in apoptotic proteins were observed by western blotting analysis, n = 3. **F** Immunofluorescence analysis of SHG-140 and T98G, cells were stained with DAPI and antibodies against BCL-2 or CLEAVED-CASPASE-3. Scale bar, 100 μm. Statistics are expressed as mean ± S.E.M., ^#^P = NS, **P < 0.01 or ***P < 0.001

Because USP7 is associated with patient prognosis and disease progression in gliomas,[27] we first focused on the expression of USP7 in GBM tissue samples. IHC analysis of tissue sections showed that USP7 expression was significantly higher in GBM than in normal brain tissue (Fig. 1A). We observed similar results using protein extracts from tissues of GBM patients and normal brains (Fig. 1B). Together, these results indicated that USP7 is highly expressed in GBM cells, which we used as a basis for targeting USP7 for GBM treatment.

Next, we focused on the biological significance of USP7 interference. Since inhibition of USP7 induces apoptosis in neuroblastoma [28], breast cancer [29] and ovarian cancer cells [30], we hypothesized that inhibition of USP7 could also induce apoptosis in GBM. We used two different interfering RNAs to transfect SHG-140 and T98G cells and western blotting analysis to examine USP7 expression. The results showed that USP7 expression significantly decreased (Fig. 1D and E). To investigate changes in apoptosis rate after transfection of SHG-140 and T98G cells with shRNAs, we used Annexin V-FITC/PI flow cytometry. As shown in the Fig. 1C, in SHG-140 or T98G, cells transfected with both shRNAs had significantly more Q2 (Annexin V-FITC + PI+), Q3 (Annexin V-FITC + PI-) and Q2 + Q3 subpopulations than the control group. This indicates that knockdown of USP7 caused an increase in early-, late-stage apoptosis and total apoptosis. We then examined the expression of apoptosis-related proteins after cell transfection. As shown in Fig. 1D and E, the expression of BCL2 was decreased and the expression of BAX and CLEAVED-CASPASE 3 was significantly increased in SHG-140 and T98G cells transfected with shRNAs compared with the control, indicating that these cells underwent significant apoptosis. Under the same treatment, we determined the expression of apoptosis-related proteins by immunofluorescence and obtained consistent results (Fig. 1F). Together, the above results demonstrated that USP7 is highly expressed in GBM, and inhibition of USP7 with interfering RNAs significantly induces apoptosis.

The research methodology for this study is shown in a flow chart (Additional file 1: Figure S1).

### The USP7 inhibitor P5091 induces apoptosis in GBM cells

**Fig. 2:**
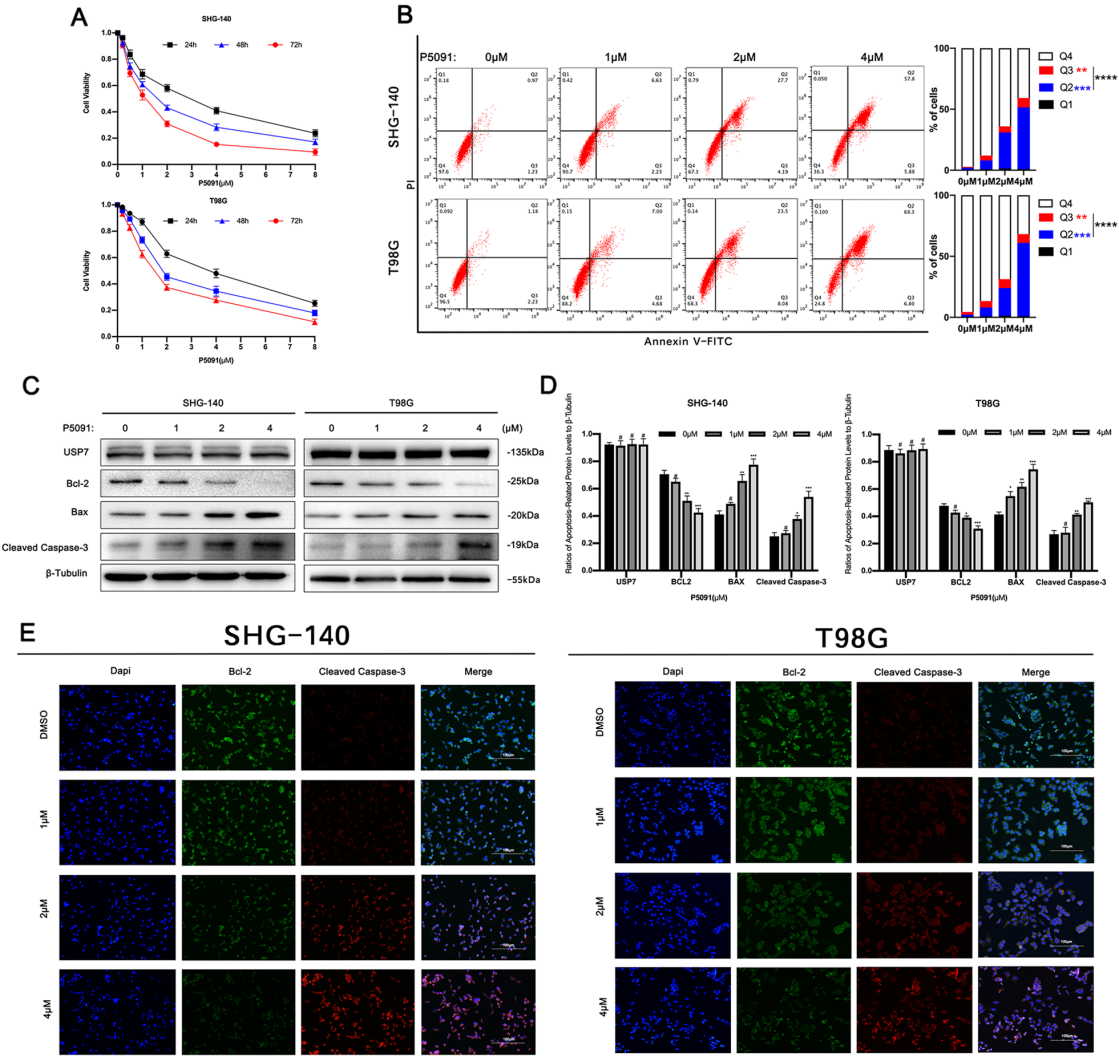
P5091 induces apoptosis in GBM cells **A** SHG-140 and T98G cells were treated with P5091 for 24, 48, and 72 h. Cell viability was determined by CCK-8 assay. **B** The apoptosis of SHG-140 and T98G cells treated with different concentrations of P5091 for 48 h was measured by flow cytometry, n = 3. Q3 (Annexin V-FITC + PI-) subpopulation was considered early-stage apoptosis and Q2 (Annexin V-FITC + PI+) was late-stage apoptosis or necrosis. Cell proportion comparisons between groups were done in both early- and late-stage subpopulation separately and in total. **C**, **D**. SHG-140 and T98G cells were treated with P5091 for 48 h. Changes in apoptotic proteins were observed by western blotting analysis, n = 3. **E** Immunofluorescence analysis of SHG-140 and T98G, cells were stained with DAPI and antibodies against BCL-2 or CLEAVED-CASPASE 3. Scale bar, 100 μm. Statistics are shown as **P < 0.01, ***P < 0.001 or ****P < 0.0001

We evaluated the effect of USP7 inhibitor P5091 on the viability of SHG-140 and T98G cells using the Cell Counting Kit-8. As shown in the results of Fig. 2A, treatment with different concentrations of P5091 for different times resulted in a significant decrease in the viability of both cell lines. The toxic effects of P5091 on SHG-140 and T98G cells were time- and concentration-dependent. The IC50 of SHG-140 and T98G cells were 1.2 µM and 1.59 µM, respectively, when treated with P5091 for 48 h. Based on IC50, SHG-140 and T98G cells were treated with P5091 for 48 h at 1, 2, and 4 µM. To explore whether P5091 induced apoptosis in GBM cells, we used Annexin V-FITC/PI flow cytometry. As shown in the Fig. 2B, as the concentration of P5091 increased, the Q2 (Annexin V-FITC + PI+), Q3 (Annexin V-FITC + PI-) and Q2 + Q3 subpopulations of both SHG-140 or T98G were significantly more than the control group. This indicates that application of P5091 caused a concentration-dependent increase in early-, late-stage apoptosis and total apoptosis in GBM cells. Similarly, we performed western blotting analysis to examine the levels of apoptosis-related proteins in SHG-140 and T98G cells treated with different concentrations of P5091 for 48 h. As shown in Fig. 2C, D, there were no significant changes in BCL2, BAX, and CLEAVED-CASPASE 3 after treatment with 1 µM P5091 for 48 h compared with the DMSO control. After treatment with 2 µM and 4 µM P5091 for 48 h, BCL2 protein expression was significantly reduced, and BAX and CLEAVED-CASPASE 3 protein expression was significantly increased in a concentration-dependent manner. These results were consistent with those of immunofluorescence (Fig. 2E). The above results indicated that P5091 has a significant pro-apoptotic effect on GBM cells, and the apoptotic effect increases with increasing concentration.

### ARF4 binds to USP7 and is downregulated by USP7 inhibition

**Fig. 3:**
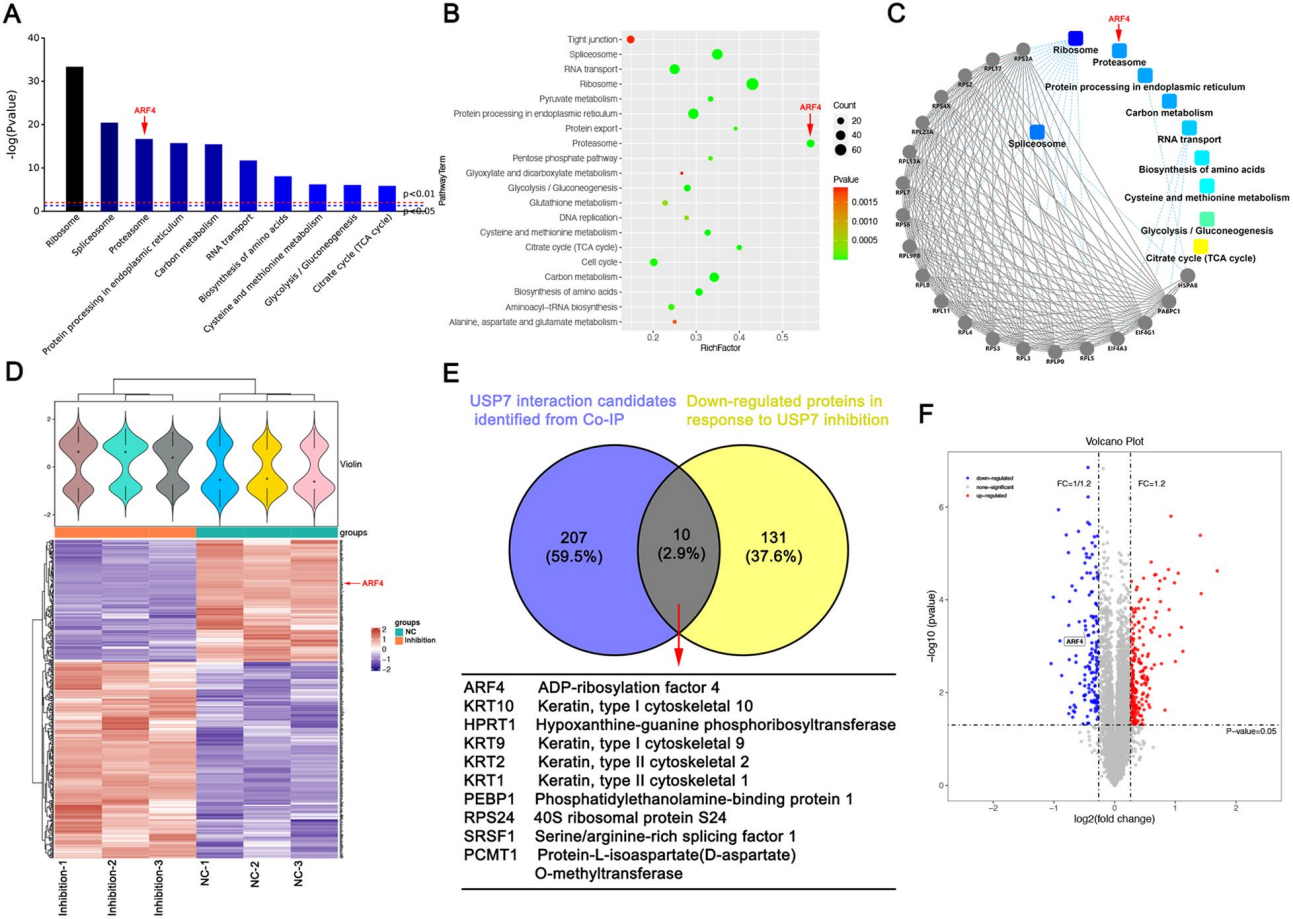
ARF4 binds to USP7 and is downregulated by USP7 inhibition **A-C** Proteins bound to USP7 were identified in SHG-140 by Co-IP and MS, and the screened proteins were analyzed for the KEGG pathway. **D** Heat map showing the comparative results of TMT proteomics analysis after 48 h treatment using DMSO or P5091 (2µM) in SHG-140. **E** Venn diagram showing down-regulated proteins in response to P5091 (yellow), USP7 interaction candidates identified by Co-IP (purple), and overlapping proteins. **F** Volcano plot showing the differential proteins from TMT proteomics analysis and the location of ARF4

To determine the mechanism by which USP7 affected apoptosis in GBM cells, we used a combination of Co-IP and LC-MS/MS to identify USP7-binding proteins in SHG-140 cells. Using this approach, 217 proteins were identified as USP7-binding proteins. We then subjected the identified proteins to KEGG pathway analysis, and Fig. 3A and B showed the top 10 and top 20 KEGG pathways in terms of significance, which were highly correlated with proteasome function. Protein interaction analysis between the identified proteins and KEGG pathways revealed that the proteasome pathway was highly significant, as shown in Fig. 3C. We then treated SHG-140 cells with DMSO and P5091 (2 µM, 48 h) and subjected the final protein extracts to TMT proteomic analysis. We found changes in the content of 368 proteins, including an increase in 227 proteins and a decrease in 141 proteins. A heat map (Fig. 3D) showed the expression of these differentially expressed proteins. Finally, we pooled and analyzed the 217 proteins identified by Co-IP with the differentially expressed protein results from proteomics, and found that 10 proteins overlapped, including ADP-ribosylation factor 4 (Fig. 3E). These proteins might be direct substrates of USP7 and their expression was altered by P5091 treatment. Here, we focused on ARF4 in view of its association with the anti-apoptotic effects of tumor cells. A volcano plot in Fig. 3F showed the downregulation of ARF4 expression in proteomic detection, and Additional file 2: Figure S2 showed the secondary mass spectra of the four peptides of ARF4. In conclusion, we successfully screened for ARF4, a protein that binds to and is affected by USP7 inhibition, and proposed the hypothesis that USP7 affects ARF4 via the proteasomal pathway.

### ARF4 is an anti-apoptotic substrate for USP7

**Fig. 4:**
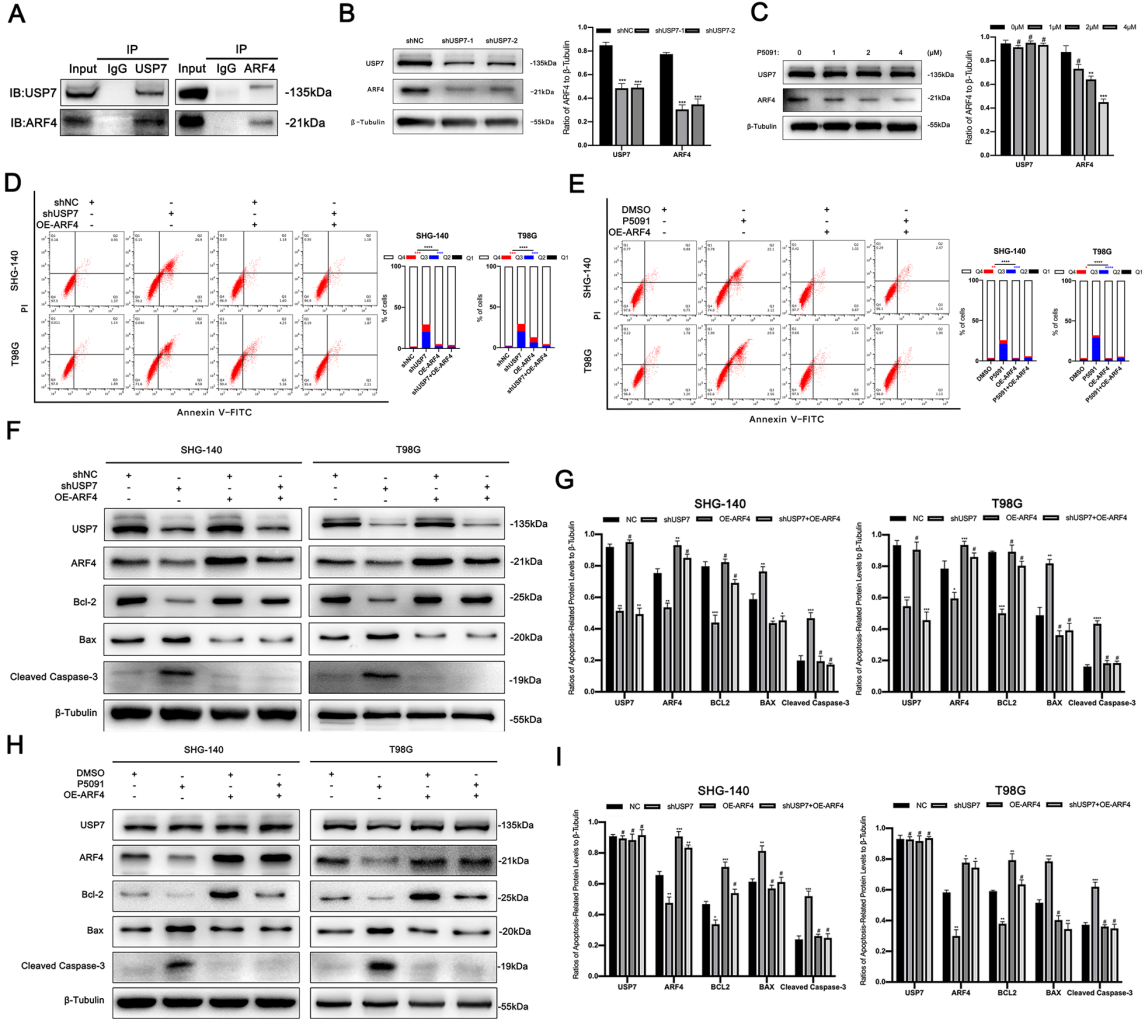
ARF4 is an anti-apoptotic substrate for USP7 **A** Interaction between USP7 and ARF4 in SHG-140 was determined using the Co-IP assay. **B** SHG-140 cells were transfected with different shUSP7 for 24 h and ARF4 expression was determined by western blotting, n = 3. **C.** Expression of ARF4 was determined by western blotting after treatment of SHG-140 with different concentrations of P5091 for 48 h, n = 3. **D**, **E** Changes in apoptosis after overexpression of ARF4 in SHG-140 and T98G cells treated with P5091 (2 µM) for 48 h or transfected with shUSP7 for 24 h were detected by flow cytometry, n = 3. Q3 (Annexin V-FITC + PI-) subpopulation was considered early-stage apoptosis and Q2 (Annexin V-FITC + PI+) was late-stage apoptosis or necrosis. Cell proportion comparisons between groups were done in both early- and late-stage subpopulation separately and in total. **F-I** Changes in apoptotic proteins after overexpression of ARF4 in SHG-140 and T98G cells treated with P5091 (2 µM) for 48 h or transfected with shUSP7 for 24 h were observed by western blotting analysis, n = 3. Statistics are expressed as mean ± S.E.M., ^#^P = NS, *P < 0.05,**P < 0.01,***P < 0.001, or ****P < 0.0001

First, we attempted to verify the relationship between USP7 and ARF4 in SHG-140. In Co-IP experiments, we found that USP7 and ARF4 binded to each other in a complex (Fig. 4A). In addition, inhibition of USP7 in SHG-140 cells using P5091 or shRNA was followed by a decrease expression in ARF4 (Fig. 4B C), which was consistent with our previous analysis. To further determine whether ARF4 was associated with apoptosis in GBM cells, we first transfected SHG-140 using two interfering RNAs for ARF4 and then used Annexin V-FITC/PI flow cytometry to measure apoptosis changes. We found that the Q2 (Annexin V-FITC + PI+), Q3 (Annexin V-FITC + PI-) and Q2 + Q3 subpopulations of SHG-140 or T98G were all significantly increased after interference with ARF4 compared to the control group, which tentatively suggests that ARF4 is associated with anti-apoptotic in GBM. (Additional file 3: Figure S3). Then we assessed the effect of ARF4 overexpression in SHG-140 and T98G cells on apoptosis in cells transfected with shRNA or after induction with P5091. We assessed the changes in apoptosis rate by Annexin V-FITC/PI flow cytometry (Fig. 4D and E). Overexpression of ARF4 in cells in which USP7 was inhibited using shRNA or P5091 resulted in a significant reduction in the Q2 (Annexin V-FITC + PI+), Q3 (Annexin V-FITC + PI-) and Q2 + Q3 subpopulations compared to the group that was inhibited alone. This suggests that ARF4 overexpression effectively prevented early-, late-stage apoptosis and total apoptosis induced by USP7 inhibition using shRNA or P5091. We then used western blotting to detect the changes in apoptosis-related proteins. As shown in Fig. 4 F–I, after ARF4 overexpression in cells under both induction conditions, no significant difference in apoptosis-related proteins was observed compared with the blank control group, and apoptosis-related proteins were significantly back-regulated compared with the intervention group. Together, these results suggested that ARF4 is an anti-apoptotic substrate for USP7 and overexpression of ARF4 counteracts apoptosis induced by targeting USP7.

### USP7 regulates ARF4 stability through K48-linked deubiquitination

**Fig. 5:**
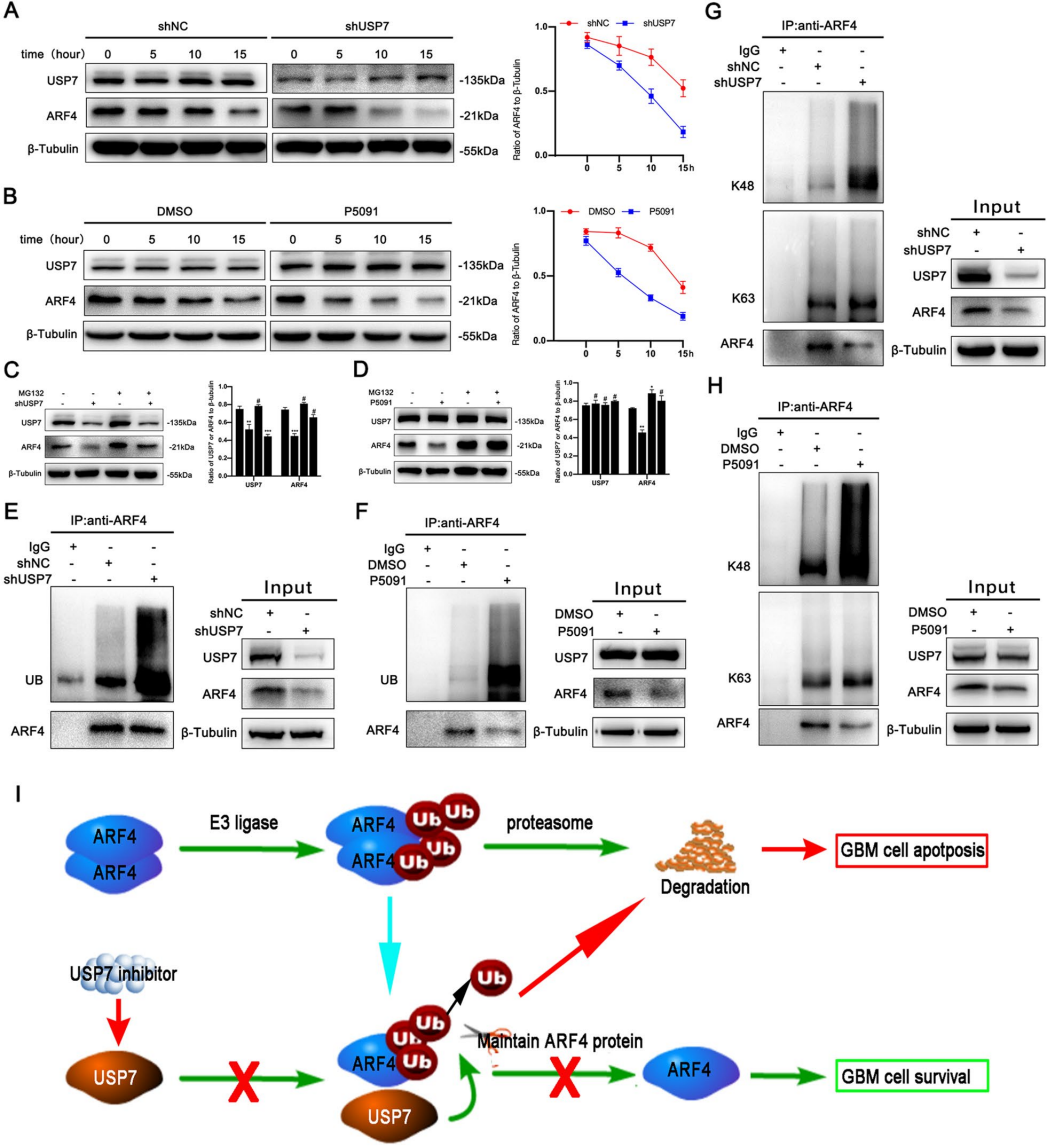
USP7 regulates ARF4 stability through K48-linked deubiquitination **A**, **B**. After transfection of SHG-140 cells with shUSP7 for 24 h or treatment of cells with P5091 (2 µM) for 48 h, cells were treated with CHX (100 µg/ml) at different times and ARF4 expression was analyzed by western blotting, n = 3. **C**, **D**. SHG-140 cells were transfected with shUSP7 for 24 h or treated with P5091 (2 µM) for 48 h followed by treatment with the proteasome inhibitor MG132 (10 µM) for 6 h. ARF4 expression was analyzed by western blotting, n = 3. **E**, **F**. SHG-140 cells were transfected with shUSP7 for 24 h or treated with P5091 (2 µM) for 48 h followed by treatment with MG132 (10 µM) for 6 h. The protein extracts were immunoprecipitated with IgG beads of anti-ARF4, and then ubiquitin and ARF4 expression were detected by western blotting. **G**, **H**. SHG-140 cells were transfected with shUSP7 for 24 h or treated with P5091 (2 µM) for 48 h followed by treatment with MG132 (10 µM) for 6 h. The protein extracts were immunoprecipitated with IgG beads of anti-ARF4, and then the expression of K48-ubiquitin, K63-ubiquitin and ARF4 was detected by western blotting. **I** A proposed mechanism for USP7 to regulate the ARF4 level in GBM. All statistics are expressed as mean ± S.E.M., ^#^P = NS, *P < 0.05,**P < 0.01 or ***P < 0.001

USP7 regulates the stability of substrate proteins through its deubiquitinating enzyme properties. Therefore, we further investigated the relationship between USP7 and the stability of ARF4. We found that interference of USP7 with P5091 or shRNA led to a decrease in the half-life of ARF4 after induction of different treatment groups at 0 h, 5 h, 10 h, and 15 h using CHX (100 µg/ml) (Fig. 5A, B). In addition, treatment with the proteasome inhibitor MG132 (10 µM, 6 h) reversed the downregulation of ARF4 after P5091- or shRNA-mediated interference of USP7 (Fig. 5C, D). This implied that USP7 maintained the steady-state level of ARF4 through the proteasome pathway, which was consistent with our previous studies. Because USP7 is a deubiquitinating enzyme, we further hypothesized that USP7 regulates the stability of the ARF4 protein through deubiquitination. To test this hypothesis, we investigated whether blocking the function of USP7 would affect ARF4 ubiquitination. We used the IP assay to assess the ubiquitination of ARF4. As shown in Fig. 5E, F, the ubiquitination activity of ARF4 was significantly increased after induction with P5091 or shRNA. This suggested that USP7 exerted a deubiquitinating effect on ARF4. Furthermore, we verified whether ARF4 ubiquitination triggered by blocking the function of USP7 was K48-ubiquitin related. We assessed the K48- and K63-ubiquitin levels of ARF4 with IP. The results showed that K48-ubiquitination of ARF4 increased after induction with P5091 or shRNA, whereas K63-ubiquitination did not change significantly (Fig. 5G, H). Together, these findings suggest that interference with USP7 decreases K48-deubiquitination of ARF4, thereby affecting the stability of ARF4 (Fig. 5I).

### P5091 inhibits tumorigenicity in intracranial xenograft models

**Fig. 6:**
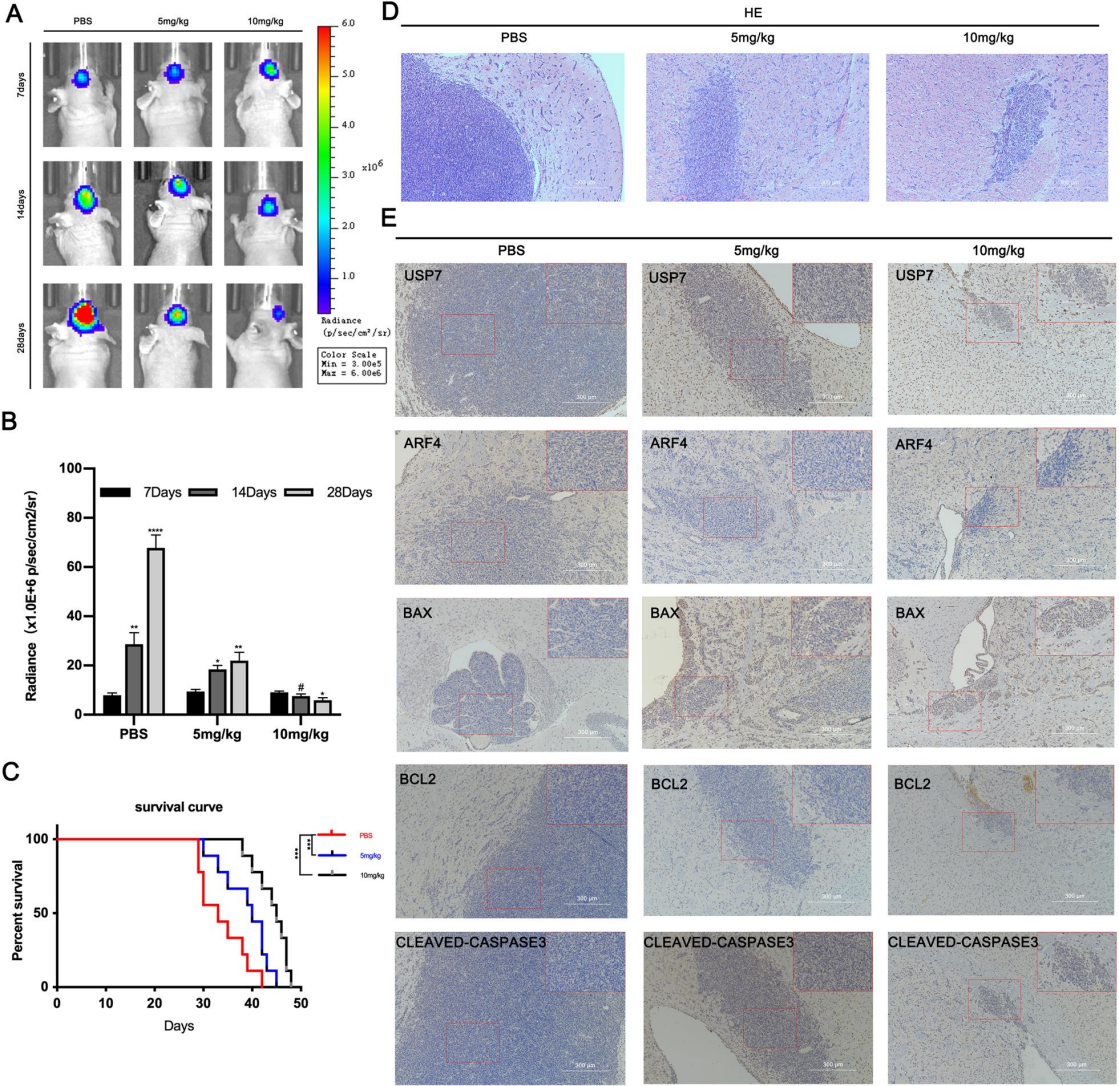
P5091 inhibits tumorigenicity in intracranial xenograft models Female BALB/c nude mice were injected intraperitoneally with PBS, P5091 (5 mg/kg/day, 10 mg/kg/day) and treated 2 days per week. Treatment started on day 7 after implantation and lasted for approximately 21 days. **A** Representative images of bioluminescence in mice on days 7, 14 and 28 after implantation. **B** Quantitative analysis of these bioluminescence images, n = 6. **C** Overall survival of the PBS and P5091 treatment groups, n = 6. **D** Representative H&E images of tumor sections. Scale bar, 300 μm. **E** Representative IHC images of tumor sections for anti-USP7, anti-ARF4, anti-BAX, anti-BCL-2, and anti-CLEAVED-CASPASE. Scale bar, 300 μm. Statistics are expressed as mean ± S.E.M., ^#^P = NS, *P < 0.05,**P < 0.01 or ****P < 0.0001

To investigate the therapeutic effect of P5091 on GBM, we established an intracranial orthotopic xenograft model by transplanting SHG-140 cells into the brain of small female nude mice. Starting from day 7 after successful implantation, PBS, 5 mg/kg of P5091 and 10 mg/kg of P5091 were injected intraperitoneally twice a week for 3 weeks. Subsequently, we observed tumor growth by bioluminescence imaging weekly, and used the tumor size and radiographic values as indicators to assess the treatment effect. We found that the growth rate of intracranial transplanted tumors in mice after the application of P5091 treatment was significantly lower than that of the blank control group (Fig. 6A, B). The survival time of mice treated with P5091 was significantly longer than that of mice in the blank control group (Fig. 6C). In addition, H&E staining of brain sections from nude mice showed significant differences in tumor growth after treatment with P5091 (Fig. 6D). IHC staining of brain sections showed that the expression of apoptosis-related proteins BAX and CLEAVED-CASPASE 3 was elevated and expression of BCL2 and ARF4 was decreased, in the treated group, whereas the expression of USP7 did not differ significantly from that in the PBS group (Fig. 6E). The in vivo experiments yielded similar results to the in vitro experiments, in which P5091 inhibited the tumorigenicity of GBM and eventually caused an increase in the expression of apoptosis-associated proteins. This suggests that P5091 has a great potential for application in clinical treatment.

## Discussion

GBMs are highly resistant to current therapeutic approaches [31]. Therefore, the discovery of other effective antitumor therapies may provide additional options for refractory GBM. In this study, we confirm the role of USP7 as a new therapeutic target for GBM. The balance between ubiquitination and deubiquitination plays an important role in the homeostasis of the cellular protein pool [32]. USP7 is involved in post-translational modifications that mediate protein stability and protein function [33]. However, the role of USP7 in cancers is paradoxical. For example, Li et al. showed that USP7 stabilizes the p53 protein and acts as a tumor suppressor through deubiquitination [34]. In contrast, a study by Cummins et al. found that another target substrate of USP7 is an E3 ligase, MDM2, and that MDM2 also has a regulatory effect on P53, which ultimately leads to the degradation of 53 and acts as an oncogene [14].This suggests that USP7 can act as an oncogene or a tumor suppressor gene depending on the role played by its protein substrate and the amount of its own expression levels. In non-small cell lung cancer (NSCLC) studies, possibly due to a different genetic background, USP7 is considered to be a tumor suppressor [35]. In our study, we provide strong evidences for the identification of USP7 as an oncogene in GBM. We tentatively reached this conclusion by observing apoptosis induced by the application of the suppression system of USP7. We found that the use of both interfering RNA and drugs that inhibit USP7 can cause apoptosis in GBM cells. Of interest, it has also been suggested that inhibition of USP7 inhibits the migration and invasion of cancer cells [36]. However, whether this occurs in GBM cells is not known, and we will continue to explore other changes in cell behaviour following inhibition of USP7 in future studies. In gliomas, expression of USP7 is positively correlated with disease progression and poor patient survival [27]. But the relationship between USP7 and patient survival in GBM remains unclear and we will attempt to analyze this in future studies using Cox proportional hazard regression and random survival forest (RSF) [37, 38].

In cancer research, the use of genomic [39, 40] and proteomic [41] approaches allows for a more accurate identification of target genes and target proteins. In hepatocellular carcinoma, Cai et al. screened thyroid hormone receptor-interacting protein 12 (TRIP12) as a substrate of USP7 by Co-IP and proteomics [42]. We filtered ARF4 in GBM in the same way. The significant reduction in the half-life of ARF4 observed after blocking the deubiquitination of USP7 and the detection of more ARF4 expression in cells after inhibition of proteasome activity suggests that USP7 regulates the stability of ARF4 by deubiquitination. It is generally believed that proteins labeled by Lys-48-linked Ub chains regulate protein stability through proteasomal degradation, whereas proteins labeled by Lys-63-linked Ub chains perform nonproteolytic processes such as DNA repair and signal transduction [43, 44]. We hypothesized and verified the conjecture that USP7 mediates the stability of ARF4 by removing the Lys-48-linked Ub chain, but not the Lys-63-linked Ub chain. ARF4 was considered an anti-apoptotic factor because ARF4 inhibits BAX-induced apoptosis in yeast [45]. It is thought that the anti-apoptotic effect of ARF4 may be related to the inhibition of ROS production [25]. In our study, we confirmed the anti-apoptotic effect of ARF4 on GBM cells. Meanwhile, we found that overexpression of ARF4 in GBM caused changes in BCL-2 family apoptosis-related proteins such as BCL-2 and BAX, suggesting that ARF4 is likely involved in BAX-mediated mitochondria-associated apoptosis. As both BCL-2 and BAX were significantly altered, we speculate that ARF4 may be a promoter of BCL-2 or a suppressor of BAX, which requires further experimental exploration in the future.

In our study, we document the therapeutic role of P5091 in GBM and affirm the potential to develop it as a new targeted agent. P5091 was originally identified as a specific inhibitor of USP7 by Chauhan et al. through a high-throughput screen [46]. P5091 acts as a covalent inhibitor of USP7, which covalently binds to the C223 residue of the catalytic domain of USP7, ultimately blocking its interaction with Ub to achieve anti-deubiquitination [47]. Although covalent inhibitors lack selectivity over non-covalent inhibitors, P5091 is not only highly specific but also irreversible [48]. GBMs underwent significant concentration-dependent apoptosis through effective induction. This suggests that, as in other malignancies, P5091 can also induce GBM cell death. In in vivo experiments, among numerous small molecule inhibitors of USP7, P5091 was shown to be effective in treating tumors in vivo with little to no toxicity [49, 50]. Based on the fact that genetic deletion of USP7 in mice leads to early embryonic death between embryonic day 6.5 and 7.5, we believe that the application of P5091 is safer compared to knocking down USP7 [46]. In our in vivo model, mice treated with the application of P5091 (5 mg/kg, 10 mg/kg) on a 3-week schedule showed no significant health problems. This is a preliminary indication of the potential of developing targeted drugs based on P5091.

However, this study has several limitations. Additional clinical specimens should be collected to further validate our results. Moreover, the efficacy and safety of P5091 for the treatment of GBM still need to be determined and the exact the mechanism of ARF4 in GBM apoptosis was not explored. These studies are of great importance in the future.

## Conclusions

Our observations support a model in which upregulation of USP7 in GBM leads to dysregulation of ARF4. Targeted inhibition of USP7 enhances the ubiquitination of ARF4 and ultimately mediates the apoptosis of GBM cells. In a future clinical sense, P5091 as a novel specific inhibitor of USP7 may be an effective approach for the treatment of GBM.

## Supplementary Information

**Additional file 1: Figure S1**. Flow chart depicting the research methodology used in this study.

**Additional file 2: Figure S2**. The secondary mass spectra of the four peptides of ARF4.

**Additional fiel 3: Figure S3**. Apoptosis rates of SHG-140 and T98G cells transfected with siARF4s were measured by flow cytometry, n=3. Q3 (Annexin V-FITC+PI-) subpopulation was considered early-stage apoptosis and Q2 (Annexin V-FITC+PI+) was late-stage apoptosis or necrosis. Cell proportion comparisons between groups were done in both early- and late-stage subpopulation separately and in total. Statistics are expressed as mean±S.E.M, **P<0.01 and ***P<0.001

## Data Availability

The datasets generated and/or analyzed during the current study are available in The Cancer Genome Atlas (TCGA) data portal (https://tcga-data.nci.nih.gov/tcga/) and Gene Expression Omnibus(GEO) database (https://www.ncbi.nlm.nih.gov/geo/). We hereby undertake that all data and materials are available.

## Abbreviations

GBMs: Glioblastomas
USP7: Ubiquitin-specific protease 7
ARFs: ADP-ribosylation factors
ARF4: ADP-ribosylation factor 4
Ub: Ubiquitin
DUBs: Deubiquitinating enzymes
TRIM22: Tripartite motif 22
FBS: Fetal bovine serum
H&E: Hematoxylin and eosin
TMT: Tandem mass tags
IP: Immunoprecipitation
MS: Mass spectrometry
NSCLC: Non-small cell lung cancer
