# Supplementary figures and images for: USP7 inhibition induces apoptosis in glioblastoma by enhancing ubiquitination of ARF4
Source: Cancer Cell Int. 2021 Sep 23;21:508. doi: 10.1186/s12935-021-02208-z (PMC8461901; doi:10.1186/s12935-021-02208-z)

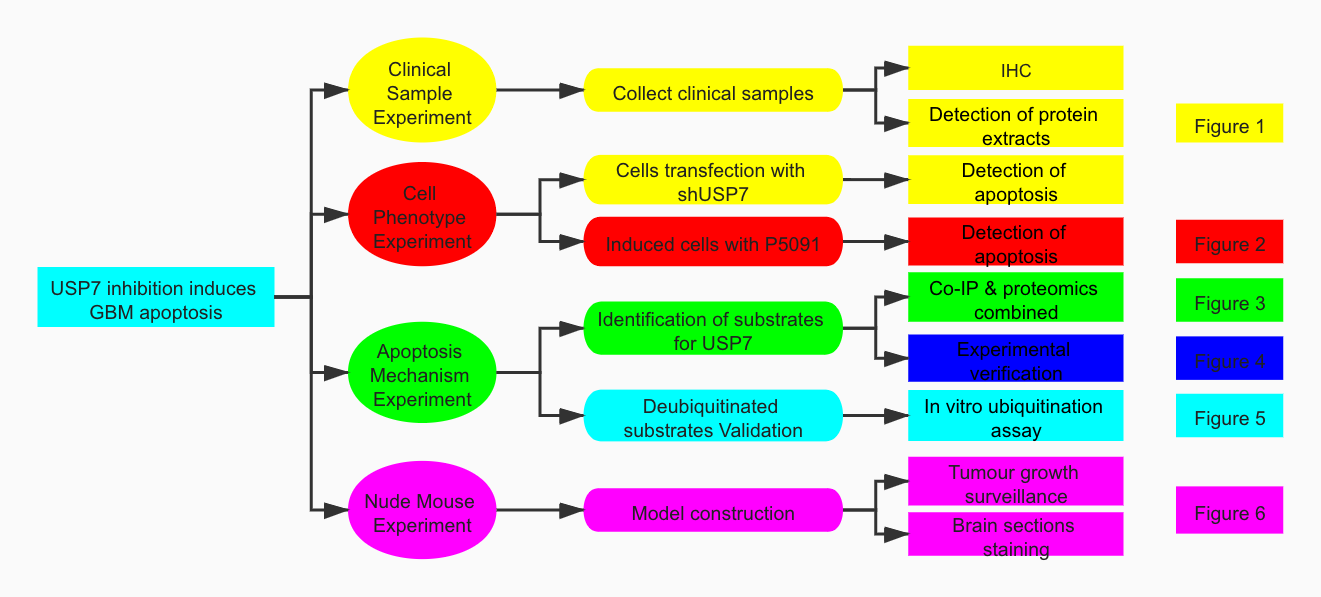

Supplement: Supplementary file 1 — Additional file 1: Figure S1. Flow chart depicting the research methodology used in this study. [file 12935_2021_2208_MOESM1_ESM.jpg]

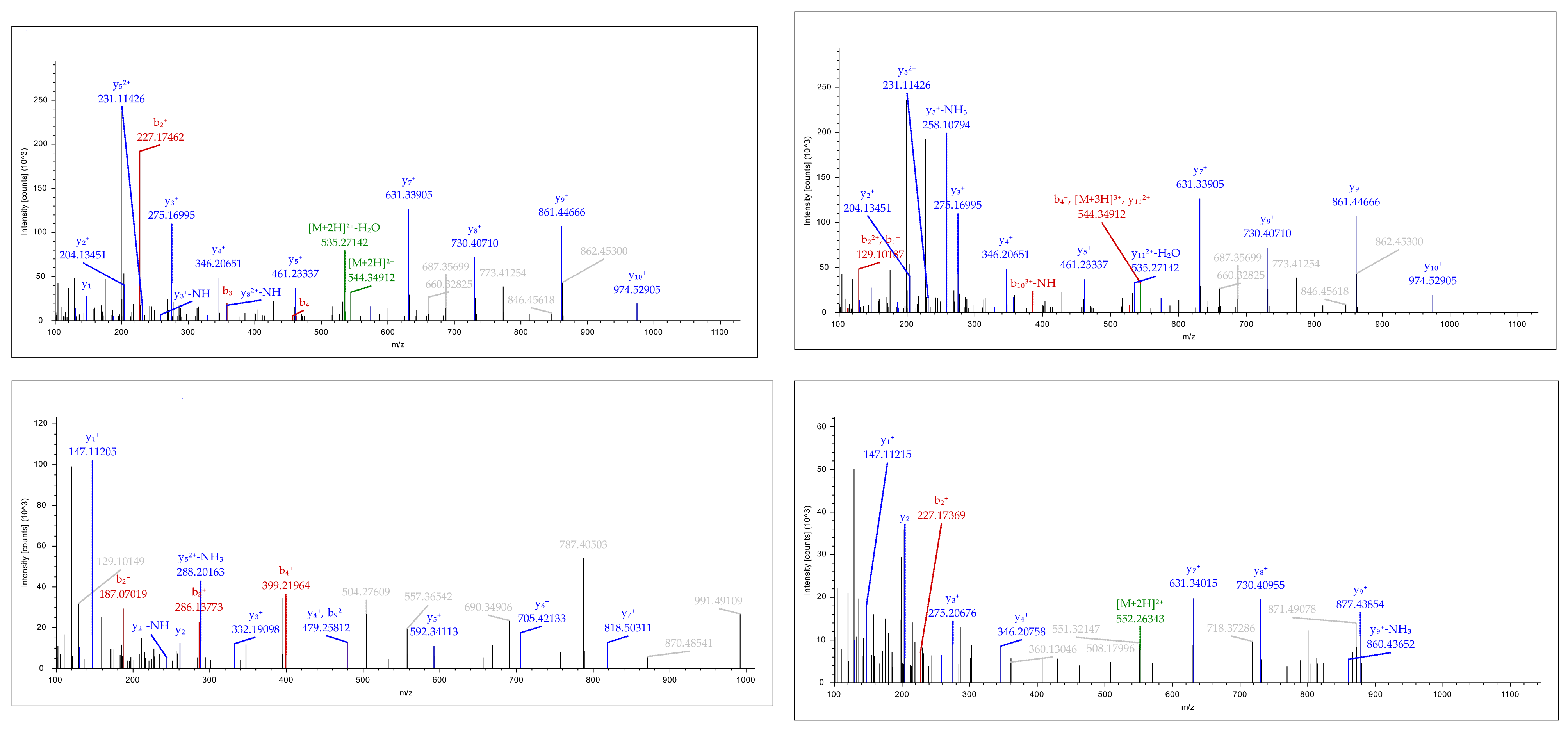

Supplement: Supplementary file 2 — Additional file 2: Figure S2. The secondary mass spectra of the four peptides of ARF4. [file 12935_2021_2208_MOESM2_ESM.tif]

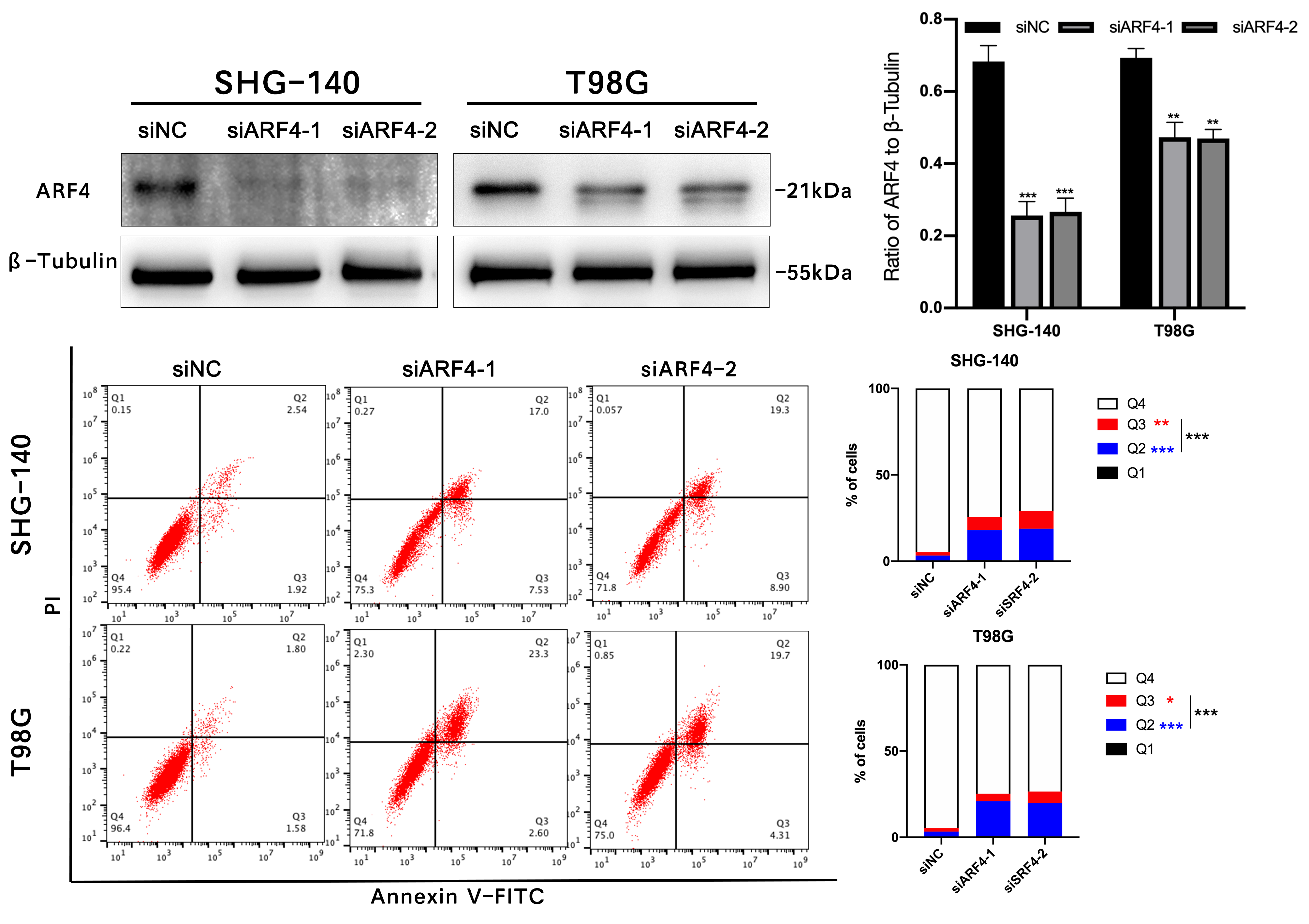

Supplement: Supplementary file 3 — Additional fiel 3: Figure S3. Apoptosis rates of SHG-140 and T98G cells transfected with siARF4s were measured by flow cytometry, n=3. Q3 (Annexin V-FITC+PI-) subpopulation was considered early-stage apoptosis and Q2 (Annexin V-FITC+PI+) was late-stage apoptosis or necrosis. Cell proportion comparisons between groups were done in both early- and late-stage subpopulation separately and in total. Statistics are expressed as mean±S.E.M, **P<0.01 and ***P<0.001 [file 12935_2021_2208_MOESM3_ESM.tif]
